# Acute administration of MK-801 in an animal model of psychosis in rats interferes with cognitively demanding forms of behavioral flexibility on a rotating arena

**DOI:** 10.3389/fnbeh.2015.00075

**Published:** 2015-04-01

**Authors:** Jan Svoboda, Anna Stankova, Marie Entlerova, Ales Stuchlik

**Affiliations:** ^1^Institute of Physiology of the Czech Academy of SciencesPrague, Czech Republic; ^2^Laboratory of Neurobehavioral Studies, National Institute of Mental HealthKlecany, Prague, Czech Republic

**Keywords:** cognitive flexibility, reversal, set-shifting, schizophrenia, MK-801, rat, Carousel

## Abstract

Patients with schizophrenia often manifest deficits in behavioral flexibility. Non-competitive NMDA receptor antagonists such as MK-801 induce schizophrenia-like symptoms in rodents, including cognitive functions. Despite work exploring flexibility has been done employing behavioral paradigms with simple stimuli, much less is known about what kinds of flexibility are affected in an MK-801 model of schizophrenia-like behavior in the spatial domain. We used a rotating arena-based apparatus (Carousel) requiring rats to avoid an unmarked sector defined in either the reference frame of the rotating arena (arena frame task, AF) or the stationary room (room frame task, RF). We investigated behavioral flexibility in four conditions involving different cognitive loads. Each condition encompassed an initial (five sessions) and a test phase (five sessions) in which some aspects of the task were changed to test flexibility and in which rats were given saline, 0.05 mg/kg or 0.1 mg/kg MK-801 thirty minutes prior to a session. In the first condition, rats acquired avoidance in RF with clockwise rotation of the arena while in the test phase the arena rotated counterclockwise. In the second condition, rats initially acquired avoidance in RF with the sector on the north and then it was reversed to south (spatial reversal). In the third and fourth conditions, rats initially performed an AF (RF, respectively) task, followed by an RF (AF, respectively) task, testing the ability of cognitive set-shifting. We found no effect of MK-801 either on simple motor adjustment after reversal of arena rotation or on spatial reversal within the RF. In contrast, administration of MK-801 at a dose of 0.1 mg/kg interfered with set-shifting in both conditions. Furthermore, we observed MK-801 0.1 mg/kg elevated locomotion in all cases. These data suggest that blockade of NMDA receptors by acute system administration of MK-801 preferentially affects set-shifting in the cognitive domain rather than reversal.

## Introduction

Adaptive response to changes in stimulus–reinforcement contingencies entails abandoning of a previous strategy, and adopting and maintaining a new efficient one. This ability is referred to as behavioral flexibility—since it often includes cognition, as cognitive flexibility. The concept of behavioral flexibility apparently covers a fairly broad spectrum of processes. At least two classes of flexibility have been recognized and regularly assessed in experimental research on rodents. Set shifting requires attendance to previously disregarded stimuli while reversals require making new associations between stimulus and the reinforcement within the old set of stimuli (Floresco et al., 2009).

Patients with schizophrenia manifest a variety of symptoms; one of the most common is the disruption of executive functions, including behavioral flexibility (Morice, 1990; Pantelis et al., 1999; Orellana and Slachevsky, 2013). Several rodent models have been established to mimic some aspects of schizophrenia-like behavior. Following the glutamate hypofunction theory of schizophrenia (Olney and Farber, 1995), models using the NMDA antagonists such as PCP, ketamine and MK-801 have been introduced successfully mimicking particular cognitive deficits (Goff and Coyle, 2001). Application of MK-801 has shown a substantial face validity (van der Staay et al., 2011), i.e., the ability to induce comparable symptoms. In terms of behavioral flexibility, its application relatively reliably simulates the data from human patients who have shown impairment both in attentional set-shifting (Pantelis et al., 1999) and reversal learning (Murray et al., 2008); thus, rats treated with MK-801 have shown disrupted reversal (van der Meulen et al., 2003; Beninger et al., 2009; Lobellova et al., 2013) (but see de Bruin et al., 2013) and set-shifting (LaCrosse et al., 2015). However, various application regimes, such as chronic, subchronic, acute or developmental administration of the drug make the rodent data difficult to interpret (Floresco et al., 2009; Amitai and Markou, 2010). Furthermore, the behavioral tests usually include a simple stimuli discrimination as in intra-dimensional/extra-dimensional shift tasks (Birrell and Brown, 2000) or 5 choice reaction task while testing flexibility during more complex problems hase been rare. Here we present a direct comparison of the effect of acute administration of MK-801 on three types of behavioral flexibility, varying in degree of cognitive demands. We employed a Carousel to accomplish this aim.

The Carousel paradigm consists of a rotating circular metal arena, in which a rat is required to avoid a particular place where it gets a mild footshock (Cimadevilla et al., 2001; Wesierska et al., 2005). Slow rotation of the arena dissociates the environmental stimuli into two distinct, coherent subsets, anchored either to the stable reference frame of the experimental room or to the rotating surface of the arena. In principle, two variants of the task based on relevancy of the corresponding reference frame can be employed (Wesierska et al., 2005). The to-be-avoided sector can be defined in the room frame (RF task) and rats must follow the set of room-stable landmarks such as the room geometry, shelves, door, posters etc. (while disregarding arena-based stimuli) to succesfully locate the sector. Alternatively, the punishment sector can be defined in the arena frame (AF task), requiring the rats to organize their spatial behavior according to the arena-based cues (scent marks, haptic cues), including the cues generated by their motion (idiothesis). In either case, rats must adopt a strategy based on one set of stimuli while abandoning the other set. To assess cognitive flexibility we used the four following conditions. (1) Training rats in the RF task, then reversing rotation includes a simple motoric response switch. (2) Training rats in the RF task, first with the sector on the north, and then switched to the south, is an example of reversal. (3, 4) Training rats in the RF task and a following switch to the AF task (or AF task followed by RF task) can be regarded as set-shifting. The Carousel apparatus provides an appropriate tool for assessing several types of flexibility at once, within a single paradigm, hence with the same kind of reinforcement and the same stimuli available, allowing exploration of behavioral flexibility while isolating other factors.

## Materials and Methods

### Animals

Ninety six adult male Long–Evans rats obtained from the breeding colony of the Institute of Physiology, ASCR were used in the study (290–350 g upon delivery, 3 months of age). They were randomly assigned to one of the four experimental conditions, each comprising three experimental groups (saline, MK-801 0.05 mg/kg, MK-801 0.1 mg/kg) with eight animals in each group. One rat of MK-801 0.05 mg/kg group from the motor switch condition was excluded from the experiment due to infection. The animals were housed in random groups of two or three per cage regardless of MK-801 treatment in a temperature-controlled animal room (21°C) with a 12/12 h light/dark cycle (lights on at 7:00). Water and food were freely available until 2 days prior to the habituation when the rats were food-restricted and maintained at 85–90% their natural body weight. A day before the start of the behavioral experiments, awake animals were gently implanted with a subcutaneous stainless needle connector, which pierced the skin between rat’s shoulders. The needle had a blunted and swirled end, which provided purchase for an alligator clip connecting a shock-delivering wire. Just prior to each session, a rat was put on a latex harness stretched from forepaws across shoulders, carrying light-emitting diode serving to locate the position of the rat. All animal manipulations were conducted in accordance with the Animal Protection Code of the Czech Republic and corresponding directives of the European Community Council (2010/63/EC). Experiments were approved by the local Animal Care Committee of the Institute of Physiology of the Czech Academy of Sciences.

### Drugs

(+)MK-801 (dizocilpine hydrogen maleate; obtained from SigmaAldrich, CR) was dissolved in sterile saline at concentrations of 0.05 and 0.1 mg/ml. Fresh solutions were prepared on the day of injection and stored in a refrigerator between injections.

### Apparatus

**Figure 1:**
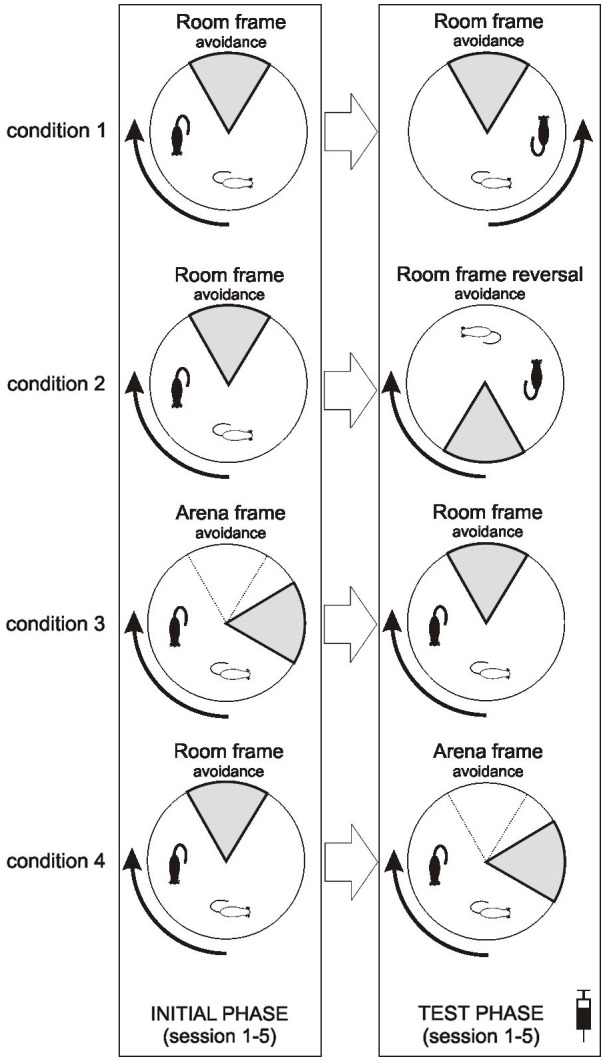
**Scheme of behavioral protocol of all four conditions employed**. Note that injections (saline or MK-801) were applied only prior to sessions in the test phase. To indicate mutual relationship of the sector and the rotating arena in each condition, position of an immobile rat and the to-be-avoided sector is shown for a time *t* at the beginning of the session (thin outlines) and *t* + 15 s (bold outlines). Note that the position of the sector remains fixed during the Room frame avoidance but rotates during the Arena frame avoidance.

The Carousel apparatus was a smooth metallic arena (82 cm in diameter), enclosed with a 30-cm-high transparent Plexiglas wall and elevated 1 m above the floor. The extra-apparatus landmarks (door, posters, and shelves) as well as the intra-apparatus cues (three fridge magnets, different in color and shape attached to the arena) were the same in all experimental conditions. At the beginning of each session, a rat was placed in the center of the arena, in which the initial constellation of the intra and extra-apparatus visual cues was held constant for all experimental conditions, thus making the RF- and AF-based cues overlap at the start of each session. Once the animal was left on the arena, the arena started to rotate constantly at one revolution per minute. An unmarked 60-degree to-be-avoided sector was defined according to the experimental condition either in the coordinate frame of the room or the arena. In the RF task, the sector location remained fixed with respect to the room coordinates and thus the rat had to use the stable extra-apparatus landmarks. On the other hand, in the AF task, the sector followed rotation of the arena, so that the rat had to rely only on the intra-apparatus cues cues combined with idiothesis (see also Figure 1). The sector was defined by a computer-based tracking system (Tracker, Biosignal Group, USA), which recorded the position of the rat (indicated by an infrared light emitting diode that was between rat’s shoulders fastened on a latex harness) at a sampling rate of 25 Hz. Another infrared diode, placed on the periphery of the arena, indicated arena rotation. The trajectories were digitized and recorded on a PC, allowing off-line reconstruction and analysis of the animal’s trajectory (Track Analysis, Biosignal Group, USA) both in the coordinate frame of the room and in the coordinate frame of the rotating arena. Whenever a rat entered the sector for more than 300 ms, constant-current regulated electric footshocks (AC, 50 Hz, 0.2–0.6 mA) were delivered at 1200-ms intervals until the rat left the sector. The shocks were administrated through the above-described subcutaneous needle connector implanted on the back of the rat standing on the grounded floor. Since the highest voltage drop of the current passing through the rat was at the high-impedance contact between the paws and the metal floor, the rats presumably perceived the shocks in their paws. The appropriate current was individualized for each rat in order to elicit a rapid escape reaction but prevent freezing (fear-related immobility). To elicit sufficient locomotor activity, which is a prerequisite for meaningful evaluation of the arena-frame avoidance, animals were diet-restricted and searched for barley grains randomly dispersed from a feeder (approx. one grain per 10 s) mounted over the arena. For comparative reasons, food restriction and food-elicited motivation was also applied during the RF avoidance condition although it was not necessary since rats were passively transported to the punished sector by rotation of the arena.

### Behavioral Training

Prior to any experimental manipulation, the rats were handled for 5 min each for 2 consecutive days. They were further habituated to the Carousel, freely moving for two daily 20 min sessions to search for randomly scattered barley grains. The study consisted of four conditions, each encompassing five sessions of an initial phase (acquisition) and five sessions of the test (of flexibility) phase (Figure 1). All sessions lasted 20 min and were conducted daily, including weekends. In the first condition (motoric response switch), rats initially acquired the RF avoidance (sector on the north) with the clockwise arena rotation, which was reversed to counterclockwise in the test phase while other conditions remained unchanged. In the second condition (reversal within RF), rats were first taught to avoid the RF-based sector located on the north and then its position was reversed to the south. In the third condition (set shifting: AF to RF switch), rats avoided the sector located on the north in the AF coordinates in the initial phase, while in the test phase its position was defined as the north in the RF coordinates. The fourth condition (set shifting: RF to AF switch) was just the opposite: RF-based avoidance was followed by AF-based avoidance in the test phase. In each condition rats were assigned to three groups which were administered (1 ml/kg body weight) saline, MK-801 0.05 mg/kg, MK-801 0.1 mg/kg, respectively, thirty minutes prior to behavioral training in the test phase.

We evaluated the portion of total session time spent in the prohibited sector as a main parameter reflecting effectivity of avoidance. Furthermore, we evaluated the locomotor activity as the total path during the session (measured as a sum of linear distances between points selected every second in the coordinate frame of the arena) which reflected only the active movement excluding passive arena rotation. We also explored the level of thigmotaxis (the amount of time spent within 16 cm from the arena wall) to quantify possible change of trajectory pattern. If the data displayed was skewed (non-normal distribution), we transformed the values with a common logarithm. Prior to this transformation, a constant of “1” was added to all values to ensure that the resultant values were not less than zero. Learning curves from the whole initial phase or the test phase were analyzed with a two-way ANOVA (MK-801 × SESSIONS) with repeated measures on sessions. Groups (saline, MK-801 0.05 mg/kg, MK-801 0.1 mg/kg) served as a between subject-factor. Likewise, data from the first session of test phase were split into two 10 min halves and analyzed with a two-way ANOVA (MK-801 × TIME SEGMENT) with repeated measures on the 10 min segment. A Tukey’s *post hoc* test followed the ANOVA when appropriate. Furthermore, in order to take individual differences into account, difference scores were computed, substracting values of the first test session from values of the last session of the initial phase. Due to non-normal distribution of the data, difference scores were analyzed using a non-parametric Kruskal-Wallis test, followed by a Dunn’s multiple comparison test when appropriate. Significance was accepted at *P* ≤ 0.05 in all cases. Statistical calculations were done in Statistica 8 (StatSoft, Czech Republic) and GraphPad Prism 5.01 (GraphPad software, Inc.).

## Results

Since application of MK-801 (>0.1 mg/kg) occasionally causes ataxia, hyperactivity or a sensomotor deficit (reviewed in van der Staay et al., 2011), we carefully observed the behavior of the rats after the injection and throughout the training session. We did not observe any aforementioned symptoms except for the mild hyperactivity after the dose 0.1 mg/kg in some cases, resulting in an elevated total path on the Carousel.

### Initial Phase

In each experiment, during the five sessions of the initial phase, rats readily acquired the “to avoid” sector; the effect of sessions conducted on the portion of time spent in the sector was significant in all cases while there was no effect of group.

#### Motor Response Switch

**Figure 2:**
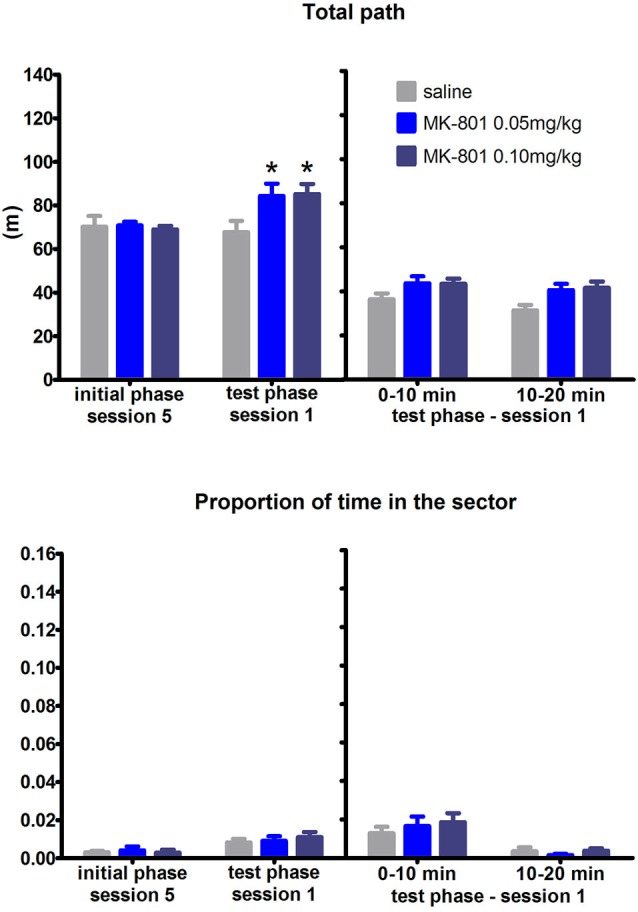
**Performance of the rats in condition 1 (motor response switch)**. Upper graph indicates total path elapsed during the last session of the initial phase, first session of the test phase, and its split into two 10 min segments (group means ± SEM). Lower graph shows proportion of session time spent in the prohibited sector. Difference between the session 1 of the test phase and the session 5 of the initial phase were computed as difference scores and the resulting statistics is provided. * *P* < 0.05 compared to saline group.

Reversing the arena rotation in the test phase of the RF task prompted rats to adjust their trajectories: instead of right turns when approaching the sector, they learned to switch to left turns. One animal from the MK-801 0.05 mg/kg group exhibiting signs of infection was excluded from the experiment. Analysis of the learning curves throughout the test phase found no effect of MK-801 application on time spent in the “prohibited” sector (*F*_(2,20)_ = 0.43, *P* = 0.66) but found significant effect on locomotor activity (*F*_(2,20)_ = 6.05, *P* = 0.009). The level of thigmotaxis remained unaffected by application of MK-801 not only in this condition, but also in the other three test conditions (data not shown). Detailed analysis of the first test session assessed by difference scores (first session of the test phase—last session of the initial phase) and session split (first vs. second half of the session) did not reveal any differences between the groups in the portion of the time spent in the sector (all *P*’s > 0.05) (Figure 2) but showed significantly increased locomotion after MK-801 application (difference scores, *H*_(2,*N* = 23)_ = 13.2, *P* < 0.01; split session, *F*_(2,20)_ = 5.54, *P* = 0.012).

#### Reversal within RF

**Figure 3:**
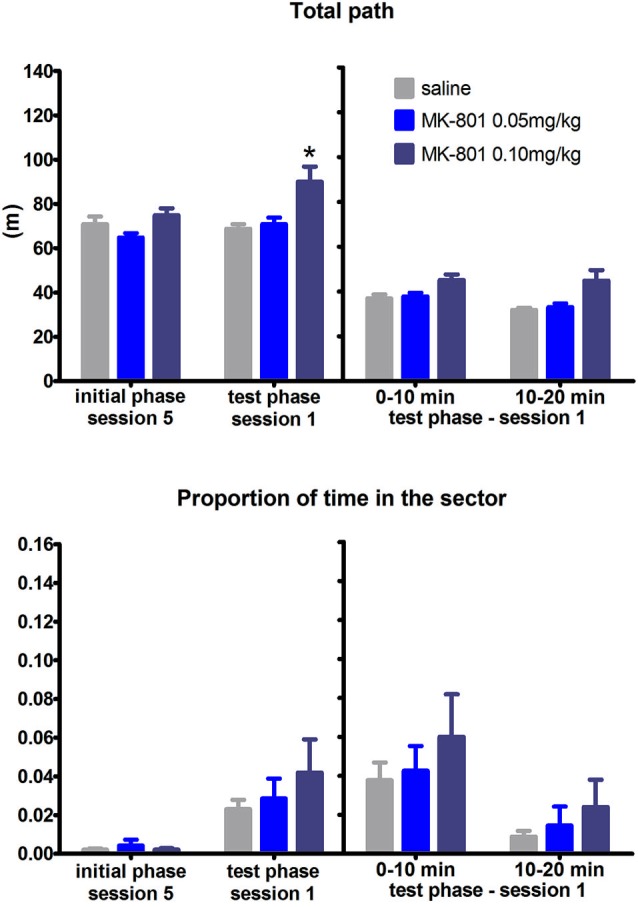
**Performance of the rats in condition 2 (reversal within RF)**. Upper graph indicates total path elapsed during the last session of the initial phase, first session of the test phase, and its split into two 10 min segments (group means ± SEM). Lower graph shows proportion of session time spent in the prohibited sector. Difference between the session 1 of the test phase and the session 5 of the initial phase were computed as difference scores and the resulting statistics is provided. * *P* < 0.05 compared to saline group.

Likewise, the ability of reversal, i.e., to relocate the sector zone from north to south within the room frame remained unaffected by MK-801 administration throughout the test phase (*F*_(2,21)_ = 0.47, *P* = 0.63). On the other hand, the rats after MK-801 application exhibited increased locomotion (*F*_(2,21)_ = 17.68, *P* = 0.023). This pattern of result was also reflected in the split session and difference scores analysis: non-significant effect of MK-801 on time spent in the sector but significant effect on the locomotion (split session, *F*_(2,21)_ = 6.94, *P* = 0.005; difference scores, *H*_(2,*N* = 24)_ = 8.42, *P* = 0.015) (Figure 3). We also evaluated the degree of perseverance. Although in principle rats do not have to necessarily abandon avoiding the previous sector (on the north) during reversal while performing avoidance of the sector on the south, we observed such perseverative behavior only within a few minutes of the reversal session. Time spent in the north location increased in the second half compared to the first half of the session (*F*_(2,21)_ = 10.76, *P* = 0.004). There was no difference between the groups.

#### Set Shifting: AF to RF Switch

**Figure 4:**
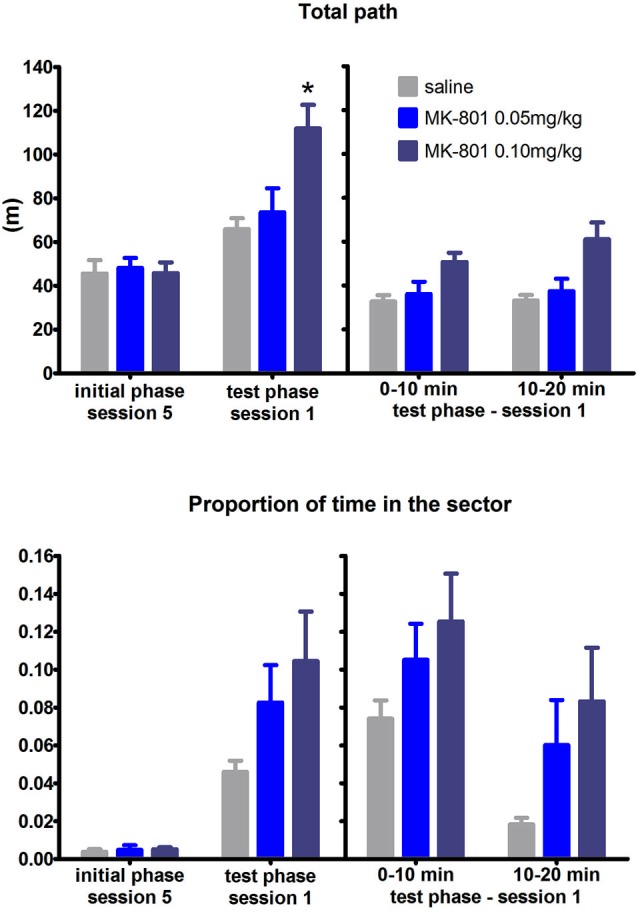
**Performance of the rats in condition 3 (set shifting: AF to RF switch)**. Upper graph indicates total path elapsed during the last session of the initial phase, first session of the test phase, and its split into two 10 min segments (group means ± SEM). Lower graph shows proportion of session time spent in the prohibited sector. Difference between the session 1 of the test phase and the session 5 of the initial phase were computed as difference scores and the resulting statistics is provided. * *P* < 0.05 compared to saline group. Difference in time spent in the sector between MK-801 0.1 mg/kg and saline group was found significant when a two-way ANOVA was conducted on the whole test phase and Tukey’s *post hoc* test applied on significant MK-801 × SESSION interaction. However, this difference was not considered significant when using difference scores.

In response to the shift from arena to the room frame coordinates all groups decreased time spent in the RF defined sector. Two-way ANOVA conducted on the whole test phase revealed significant SESSION × MK interaction (*F*_(8,84)_ = 2.31, *P* = 0.027, which was due to impaired avoidance of MK-801 0.1 mg/kg group in session 1 (Figure 4) compared to saline group (Tukey’s *post hoc* test, *P* < 0.05). However, analysis of the difference scores and the split session failed to see a significant effect of MK-801 on spatial avoidance (both *P*’s > 0.05). Effect of MK-801 on locomotion reached significance in all analysis performed: during the whole test phase (*F*_(2,21)_ = 9.1, *P* = 0.0014), the difference scores (*H*_(2,*N* = 24)_ = 11.78, *P* = 0.003), and the split session (*F*_(2,21)_ = 6.92, *P* = 0.005). Rats did not perseverate avoiding the previous sector defined in the arena frame as time spent in the previous punishment location was quite high since the beginning of the first set-shifting session and did not increase in the second half of the session compared to the first half (effect of time segment, NS; group effect, NS; data not shown).

#### Set Shifting: RF to AF Switch

**Figure 5:**
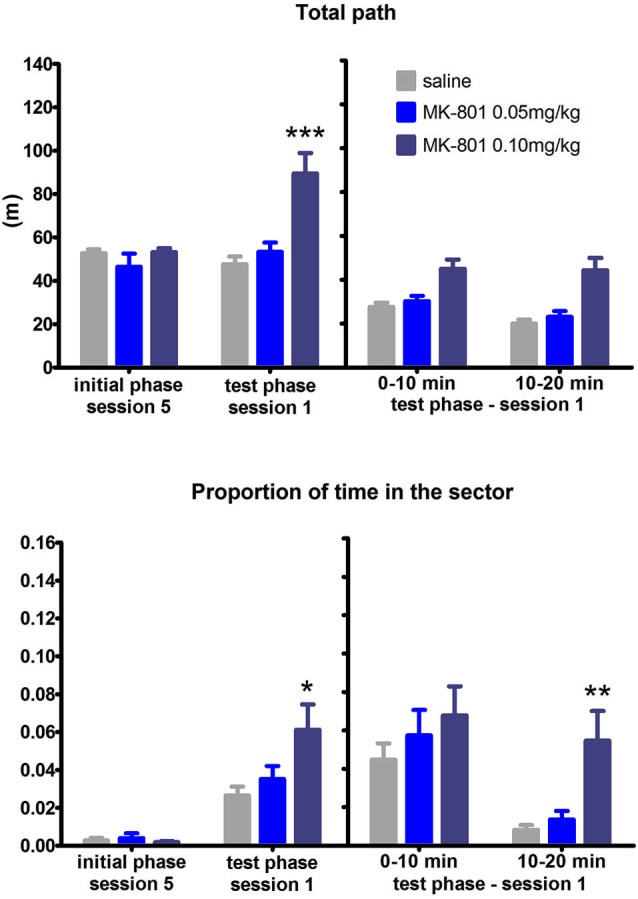
**Performance of the rats in condition 4 (set shifting: RF to AF switch)**. Upper graph indicates total path elapsed during the last session of the initial phase, first session of the test phase, and its split into two 10 min segments (group means ± SEM). Lower graph shows proportion of session time spent in the prohibited sector. Difference between the session 1 of the test phase and the session 5 of the initial phase were computed as difference scores and the resulting statistics is provided. * *P* < 0.05, ** *P* < 0.01, *** *P* < 0.001 compared to saline group.

More pronounced effect of MK-801 on the spatial flexibility was observed in the RF to AF switch condition (Figure 5). Two-way ANOVA conducted on the whole test phase found out significant SESSION × MK interaction in the time in the punishment sector (*F*_(8,84)_ = 2.44, *P* = 0.02). Tukey’s *post hoc* test revealed the MK-801 0.1 mg/kg group performed worse than the saline group in session 1 (*P* < 0.001). Moreover, there was a significant effect of MK-801 when analyzing the difference scores (*H*_(2,*N* = 24)_ = 6.1, *P* = 0.047). Split session analysis revealed that MK-801 0.1 mg/kg rats (compared to saline treated rats) spent significantly more time in the sector in the second half of the session 1 (*P* < 0.01). The effect of MK-801 on locomotion was again significant in all analysis performed: during the whole test session *F*_(2,21)_ = 3.72, *P* = 0.041, the difference scores (*H*_(2,*N* = 24)_ = 16.63, *P* = 0.0002), and the split session (*F*_(2,21)_ = 6.92, *P* = 0.003). Perseverative behavior ceased during the first session of set-shifting as time spent in location of previously punished sector was significantly higher in the second half of the session than the first half (*F*_(1,21)_ = 18.36, *P* = 0.0003). To explore the relative degree of perseveration in either set-shifting we compared the time spent in the location of previous sector for both conditions. A two-way ANOVA found no effect of MK but significant effect of condition (*F*_(1,21)_ = 10.64, *P* = 0.004), confirming that rats perseverated more after RF-AF shift than AF-RF shift.

## Discussion

We have demonstrated here that acute administration of MK-801 in the Long Evans rats at a dose of 0.1 mg/kg but not 0.05 mg/kg disrupts in particular set-shifting in the Carousel, while reversal remained almost unaffected. In addition, it did not affect any aspect of simple motoric response adjustment after reversal of rotation.

The motor response switch represents a test of simple flexibility; neither entails a change of stimulus response contingencies, nor a need to attend to a new set of stimuli. The target remains at the same position so it is still associated with the same set of (room-based) stimuli. Reversal of the rotation, however, requires the rat to adjust organizing its avoidance trajectory accordingly. Although pellets drop randomly onto the arena surface, the rat usually walks near the arena wall. When the animal approaches the punishment sector—passively by rotation or by its active movement—it quickly runs opposite to rotation direction, making a right turn just prior the run to take the shortest route to the place furhtest from the sector. If the arena rotates counterclockwise, the rat is then required to make left turns to take the appropriate shortcut. We saw that rats quickly reorganized their trajectory in response to reversal of arena rotation, with no differences among groups, despite application of MK significantly increased locomotion. MK (up to 0.1 mg/kg) therefore does not prevent flexible adjusting escape trajectories while the position of the target remains constant in the room coordinates. This kind of flexibility seems, to some extent, analogous to reversal of body responses in the T-maze which, however, was found to be easily disrupted by MK-801 application in developing rats (Chadman et al., 2006). The authors reported a selective effect of acute administration (0.06 mg/kg and 0.10 mg/kg, but not 0.03 mg/kg) on flexible reversal from right to left turns (or *vice versa*).

Reversal of target position in the room frame coordinates requires rats to retain the same set of relevant stimuli (RF based spatial cues) but to make new goal—stimulus associations. Reversal in the Carousel has been evaluated previously. Although Lobellova et al. (2013) used slightly different training protocols than our RF reversal condition, with only three sessions of acquisition and two sessions of reversal, we find the results quite comparable; they demonstrate a deficit in rats treated with MK-801 in the doses 0.08 mg/kg, 0.12 mg/kg, and 0.15 mg/kg, but not 0.10 mg/kg. Thus, the level around 0.1 mg/kg seems to be a threshold for reversal deficit, a value that has been shown also in a water maze reversal with hidden (Lobellova et al., 2013) or visible platform (Ahlander et al., 1999). Surprisingly, this level has also been recognized in simple reversals employing a Skinner box as de Bruin et al. (2013) finding no deficit in reversal from right lever to left lever pressing in Sprague Dawley rats treated with subchronic MK-801 up to doses of 0.075 mg/kg while a previous study (van der Meulen et al., 2003) revealed a higher dose (0.1 mg/kg) completely inhibited reversal learning. Subchronic pretreatment regimens exert less effect on reversals as 0.5 mg/kg MK (twice daily, 7 days) administration resulted in only mildly disrupted learning of reversed platform position in a water maze (Beninger et al., 2009). Moreover, pretreatment with a single high dose of MK-801 causes rather a procedural deficit than inflexibility in RF reversal in the Carousel (Lobellová et al., 2014). Collectively these data suggest that a reversal deficit is dose-dependent and similar across tasks employed (Carousel, Skinner box, water maze).

Set-shiftings in the Carousel require a shift of attention to stimuli anchored to a previously irrelevant frame of reference. Both AF to RF and RF to AF shifts were affected by MK-801 0.1 mg/kg although our data suggest they cannot be regarded interchangeably. Rats apparently adhere more easily to RF as they display some degree of perseveration after AF shift while they did not significantly perseverate after the AF to RF shift. This is in line with the notion that distal extramaze cues exert more control on spatial behavior and its correlate, place cells, than intramaze cues (Shapiro et al., 1997). Strong evidence suggests extradimensional set-shift disrupting after systemic administration of NMDA antagonists (Egerton et al., 2005; Stefani and Moghaddam, 2005, 2010; Rodefer et al., 2012) while there is little effect on intra-dimensional set-shift (Gastambide et al., 2012; Rodefer et al., 2012). Our set-shifting manipulations are extra-dimensional in nature. They do not obviously represent shifts in modality, but shifts of mental sets as arena based cues and room based cues are framed into two distinct and independent representations mediated by distinct neuronal populations (Fenton et al., 1998; Kelemen and Fenton, 2010). Our results from set-shifting therefore support the notion that it is easily disrupted after administration of NMDA antagonists. However, they further raise the question of what neuronal networks are involved. While the rat medial prefrontal cortex has been shown to be a crucial structure underlying set-shifting mechanisms (for review see Floresco et al., 2009; Hamilton and Brigman, 2015), neurons coordinating room frame and arena frame representations have been found in the hippocampus (Bures et al., 1997; Kelemen and Fenton, 2010). Furthermore, inactivating even one hemisphere of the hippocampus substantially disrupts RF avoidance (Cimadevilla et al., 2000). However, mPFC contribution to set-shifting in the Carousel or its linking to the hippocampus has not been investigated although our unpublished data suggest that mPFC is not crucially involved in coordinating AF or RF representations and reversal learning in RF. It is interesting that rats with induced subclinic hepatic encephalopathy show cognitive deficits including deficit in RF-AF set-shifting in Carousel (Wesierska et al., 2006).

An important issue raised inevitably in all studies using MK-801 in the Carousel is that MK-801 induced hyperlocomotion is a primary cause for elevated time spent in the punishment sector. Normalizing our data by dividing time in the sector by elapsed path did not provide convincing evidence against this concern (main effect of MK: *P* < 0.05 in all cases), which was allayed in some other experiments (Kubík et al., 2014). However, if MK-801 administration at dose 0.1 mg/kg causes hyperactivity that is not spatially organized, then such random motion would alter thigmotaxic behavior—and we did not observe this effect under any condition. In addition, elevated locomotion would also affect sector avoidance in all flexibility experiments, not only in set-shifting. For that reason we conclude that despite MK induced mild hyperlocomotion, the deficit in set-shifting is rather attributable to an inability to flexibly acquire sector location within a new set of stimuli.

The performance in the RF task itself can be affected by NMDA antagonism, but at much higher doses (0.2 mg/kg) of MK-801, which rather interferes with elevated locomotion resulting in a higher number of entrances into the to-be-avoided sector (Stuchlik et al., 2004). Wistar strain rats, however, have been found to be more sensitive in the RF task, displaying a deficit already after 0.1 mg/kg (Vales et al., 2006; Bubenikova-Valesova et al., 2008). We therefore intentionally chose doses that would not affect learning of the RF or AF task itself, but that would be near the threshold for reversal deficit found in our previous study (Lobellova et al., 2013). Indeed, none of the MK-801 groups performed continually worse than saline treated rats indicating unaffected maintenance of RF based avoidance. Impaired performance only during the first session of the test phase suggests that it is due to an inability to flexibly shift from one frame-based avoidance to another.

In general, our findings of preferential disruption in set-shifting to reversal under acute hypofunction of NMDA receptors by MK-801 treatment suggest that underlying anatomical substrate of both processes is differentially sensitive to systemic administration of MK-801.

## Conflict of Interest Statement

The authors declare that the research was conducted in the absence of any commercial or financial relationships that could be construed as a potential conflict of interest.
