# Corrigendum: Acute administration of MK-801 in an animal model of psychosis in rats interferes with cognitively demanding forms of behavioral flexibility on a rotating arena

**DOI:** 10.3389/fnbeh.2015.00348

**Published:** 2015-12-16

**Authors:** Jan Svoboda, Anna Stankova, Marie Entlerova, Ales Stuchlik

**Affiliations:** ^1^Institute of Physiology Academy of SciencesPrague, Czech Republic; ^2^National Institute of Mental HealthKlecany, Czech Republic

**Keywords:** MK-801, rats, flexibility, active place avoidance, memory

In the paper by Svoboda et al., a mistake was made in the Acknowledgment Section. The corrected Acknowledgments follow:

## Conflict of interest statement

The authors declare that the research was conducted in the absence of any commercial or financial relationships that could be construed as a potential conflict of interest.

